# A novel covalent approach to bio-conjugate silver coated single walled carbon nanotubes with antimicrobial peptide

**DOI:** 10.1186/s12951-016-0211-z

**Published:** 2016-07-13

**Authors:** Atul A. Chaudhari, D’andrea Ashmore, Subrata deb Nath, Kunal Kate, Vida Dennis, Shree R. Singh, Don R. Owen, Chris Palazzo, Robert D. Arnold, Michael E. Miller, Shreekumar R. Pillai

**Affiliations:** Center for Nanobiotechnology Research, Alabama State University, Montgomery, AL USA; Department of Mechanical Engineering, University of Louisville, Louisville, KY USA; Therapeutic Peptides Inc., 7053 Revenue Drive, Baton Rouge, LA 70809 USA; Department of Drug Discovery and Development, Auburn University, Auburn, AL USA; Research Instrumentation Facility, Auburn University, Auburn, AL USA

**Keywords:** Carbon nanotubes, Antimicrobial, Peptide, Bacteria, Cytotoxicity, Bio-conjugation

## Abstract

**Background:**

Due to increasing antibiotic resistance, the use of silver coated single walled carbon nanotubes (SWCNTs-Ag) and antimicrobial peptides (APs) is becoming popular due to their antimicrobial properties against a wide range of pathogens. However, stability against various conditions and toxicity in human cells are some of the major drawbacks of APs and SWCNTs-Ag, respectively. Therefore, we hypothesized that APs-functionalized SWCNTs-Ag could act synergistically. Various covalent functionalization protocols described previously involve harsh treatment of carbon nanotubes for carboxylation (first step in covalent functionalization) and the non-covalently functionalized SWCNTs are not satisfactory.

**Methods:**

The present study is the first report wherein SWCNTs-Ag were first carboxylated using Tri sodium citrate (TSC) at 37 °C and then subsequently functionalized covalently with an effective antimicrobial peptide from Therapeutic Inc., TP359 (FSWCNTs-Ag). SWCNTs-Ag were also non covalently functionalized with TP359 by simple mixing (SWCNTs-Ag-M) and both, the FSWCNTs-Ag (covalent) and SWCNTs-Ag-M (non-covalent), were characterized by Fourier transform infrared spectroscopy (FT-IR), Ultraviolet visualization (UV–VIS) and transmission electron microscopy (TEM). Further the antibacterial activity of both and TP359 were investigated against two gram positive (*Staphylococcus aureus* and *Streptococcus pyogenes*) and two gram negative (*Salmonella enterica* serovar Typhimurium and *Escherichia coli*) pathogens and the cellular toxicity of TP359 and FSWCNTs-Ag was compared with plain SWCNTs-Ag using murine macrophages and lung carcinoma cells.

**Results:**

FT-IR analysis revealed that treatment with TSC successfully resulted in carboxylation of SWCNTs-Ag and the peptide was indeed attached to the SWCNTs-Ag evidenced by TEM images. More importantly, the present study results further showed that the minimum inhibitory concentration (MIC) of FSWCNTs-Ag were much lower (~7.8–3.9 µg/ml with IC50: ~4–5 µg/ml) compared to SWCNTs-Ag-M and plain SWCNTs-Ag (both 62.6 µg/ml, IC50: ~31–35 µg/ml), suggesting that the covalent conjugation of TP359 with SWCNTs-Ag was very effective on their counterparts. Additionally, FSWCNTs-Ag are non-toxic to the eukaryotic cells at their MIC concentrations (5–2.5 µg/ml) compared to SWCNTs-Ag (62.5 µg/ml).

**Conclusion:**

In conclusion, we demonstrated that covalent functionalization of SWCNTs-Ag and TP359 exhibited an additive antibacterial activity. This study described a novel approach to prepare SWCNT-Ag bio-conjugates without loss of antimicrobial activity and reduced toxicity, and this strategy will aid in the development of novel and biologically important nanomaterials.

**Electronic supplementary material:**

The online version of this article (doi:10.1186/s12951-016-0211-z) contains supplementary material, which is available to authorized users.

## Background

Carbon nanotubes are well known for their wide range of applications in diverse fields, including biomedicine [1, 2]. Of relevance, single-walled carbon nanotubes (SWNTs) have been used for biomedical molecular imaging and effective drug delivery in vivo as well as in vitro [3–9]. Additionally, metallic nanocomposites of SWCNTs, especially silver coated SWCNTs (SWCNTs-Ag) have shown a remarkable antibacterial activity against gram positive as well as gram negative pathogens over the past few years [10–12]. The results from these studies are promising as there is an urgent need of developing novel antimicrobial strategies due to increasing resistance to several broad spectrum antibiotics [13–15]. However, the application of SWCNTs-Ag has been limited due to several known mechanisms of toxicity to eukaryotic cells [16–18]. Functionalization strategies have been developed and reported to lessen their toxicity, such as pegylation or surface modification using biological entities like DNA/RNA or protein [11, 12, 16–18]. In our previous study we demonstrated that pegylation of SWCNTs-Ag reduced toxicity to different eukaryotic cell lines without reducing their anti-bacterial activity [11]. Although surface modification of SWCNTs-Ag reduced their toxic effects on eukaryotic cells, the functionalization was not stable and the dosage required for the antibacterial activity remained high (62.5 µg/ml).

Functionalization using antimicrobial peptides (APs) may reduce the dosage required for the antibacterial activity of SWCNTs-Ag due to the antibacterial activity of both the components. The application of host derived or synthetic APs are becoming popular due to their effectiveness and broad range of antibacterial activity [19–23]. Several in vitro as well as in vivo models have successfully demonstrated that APs exhibit effective antimicrobial activity [23–29]. Despite these advantages, the use of APs has several disadvantages, such as enzymatic degradation leading to loss of activity, expensive to produce and instability in solution. However, a suitable delivery vehicle can mitigate these challenges by enhancing delivery of the peptides to the infected site and minimizing degradation. Carbon nanotubes have been used effectively as a formulation platform for the targeted delivery of anticancer agents, DNAs, RNAs and proteins [30–35]. Thus, functionalization of SWCNTs to produce nanotube bioconjugates for the desired application is a rational approach. In general, functionalization can be achieved by either covalent bonding or non-covalent interactions to SWCNTs [30, 31, 33, 34]. However, various covalent functionalization protocols involves harsh treatment of carbon nanotubes such as oxidation of nanotubes, addition of 1,3-dipolar cycloaddition on the nanotube sidewalls or treatment with highly concentrated acids such as HNO_3_ and H_2_SO_4_ [33, 36–38]. Although covalent chemical reactions offer stable functionalized SWCNTs, the sidewalls of the nanotubes get severely damaged in the process. In contrast, non-covalent functionalization by amphiphilic molecules either through passive absorption or by coating of the nanotube surfaces maintains the structure and optical properties of SWCNTs [33]. However, non-covalently functionalized SWCNTs are of limited use due to poor stability and issues associated with biocompatibility. Although, SWCNTs have been functionalized earlier using anti-cancer drugs, DNA, RNA or proteins [30, 32–35], the metallic nanocomposites of SWCNTs such as SWCNTs-Ag have yet to be successfully functionalized with biological molecules.

Therefore a unique strategy is required which will result in minimal damage to the structure of SWCNTs-Ag, provide maximum biocompatibility and optimum antibacterial activity with high potency (i.e., relatively low dosage). In the present study, we developed a novel approach to functionalize SWCNTs-Ag covalently using an effective antimicrobial peptide from Therapeutic Inc, TP359. The functionalized SWCNTs-Ag were further characterized by Fourier transform infrared spectroscopy (FT-IR) and transmission electron microscopy (TEM) and compared with the non-covalently functionalized SWCNTs-Ag. Further, the antibacterial activity of functionalized SWCNT-Ag (covalent as well as non-covalent) was investigated against two gram positive (*Staphylococcus aureus* and *Streptococcus pyogenes*) and two gram negative pathogens (*Salmonella enterica* serovar Typhimurium and *Escherichia coli*).

## Results

### Carboxylation of silver coated-single walled carbon nanotubes (SWCNTs-Ag)

**Fig. 1:**
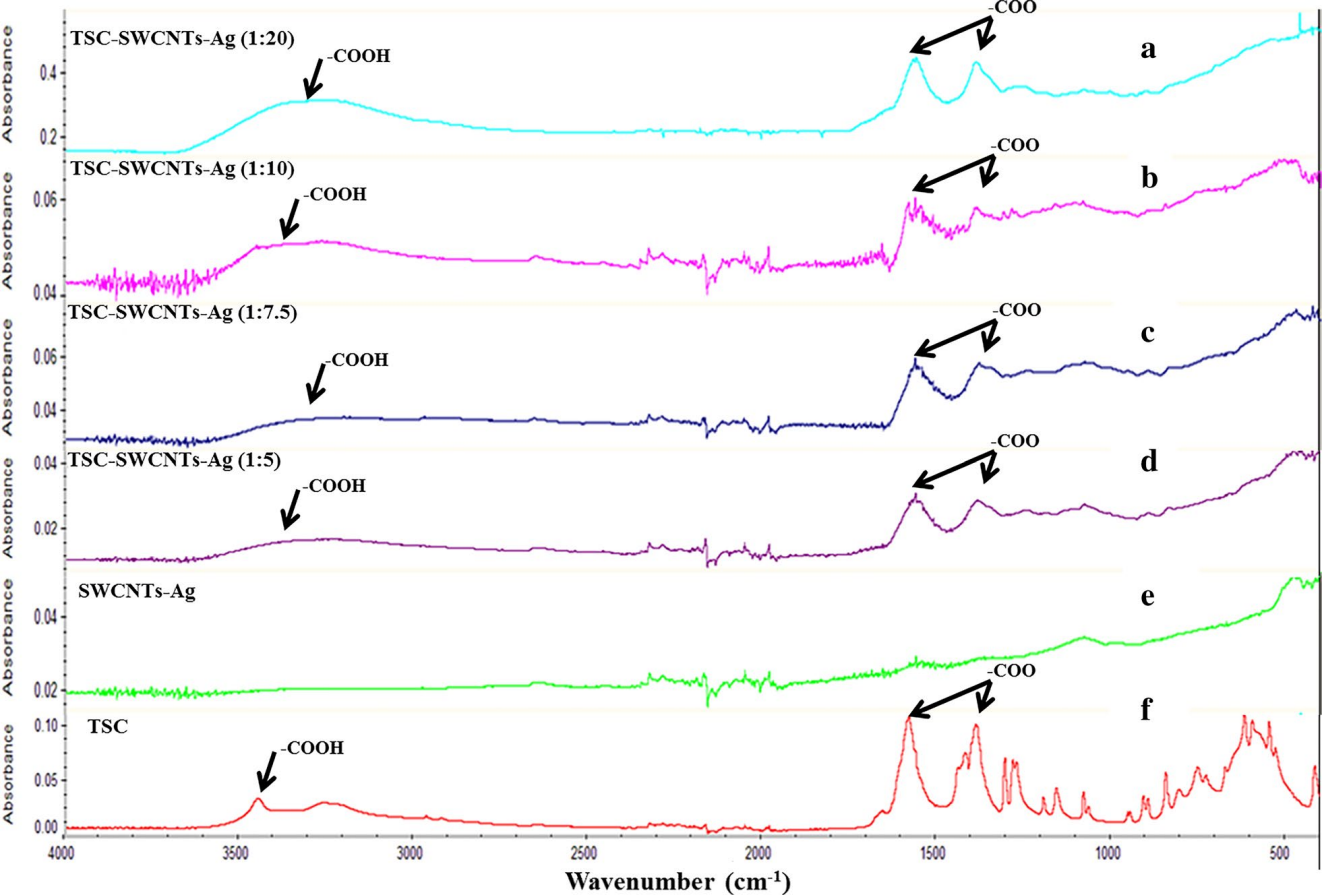
FT-IR pattern of carboxylated silver coated single walled carbon nanotubes (SWCNTs-Ag) treated with Tri-sodium citrate (TSC). The FT-IR spectra of SWCNTs-Ag treated with TSC using different ratios was investigated for the observation of the characteristic peaks related to carboxyl group. **a** TSC-SWCNTs-Ag (1:20); **b** TSC-SWCNTs-Ag (1:10); **c** TSC-SWCNTs-Ag (1:7.5); **d** TSC-SWCNTs-Ag (1:5); **e** SWCNTs-Ag; **f** TSC. The characteristic peaks on TSC and various TSC treated SWNTs-Ag such as carboxylic group stretches such as –COOH at 3300 cm^−1^ and –COO at 1300 and 1700 cm^−1^

Carboxylation on the surface of SWCNTs-Ag was confirmed by FT-IR. FT-IR spectrum of pure TSC, SWCNTs-Ag and different ratios of TSC to SWCNTs-Ag are shown in Fig. 1a–f. The characteristic absorption bands of –COO at around 1394 (symmetric stretching) and 1589 cm^−1^ (asymmetric stretching), and –COOH bands at around 3322 cm^−1^ were observed on pure TSC whereas the pure SWCNTs-Ag did not show any of these characteristic peaks on their surfaces (Fig. 1e, f). The similar peaks corresponding to the –COO and –COOH stretching were observed on TSC treated SWCNTs-Ag at different ratios (Fig. 1a–d). The presence of –COOH and –COO stretching on the TSC-SWCNTs-Ag confirmed the carboxylation of SWCNTs-Ag with the treatment of TSC.

### Covalent and non-covalent functionalization of SWCNTs-Ag with an antimicrobial peptide TP359

**Fig. 2:**
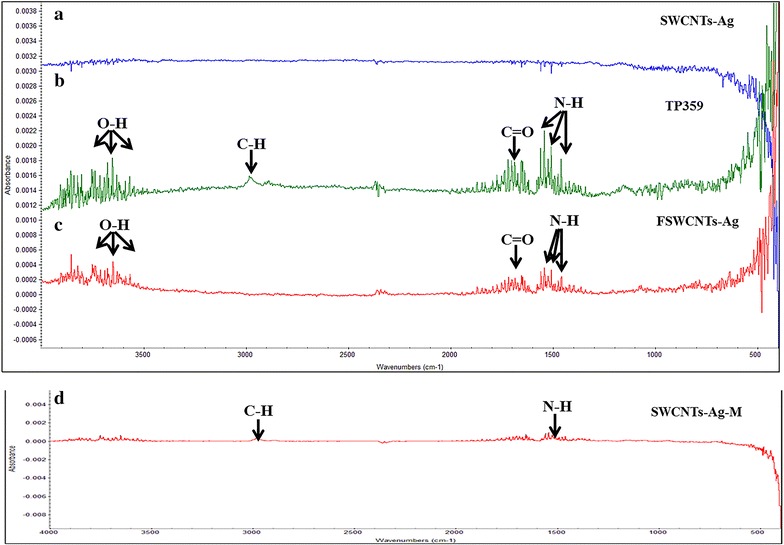
FT-IR pattern of silver coated single walled carbon nanotubes (SWCNTs-Ag) functionalized with the antimicrobial peptide TP359. SWCNTs-Ag were either carboxylated using TSC and subsequently functionalized with TP359 (FSWCNTs-Ag) or simply mixed with TP359 (SWCNTs-Ag-M) and FT-IR spectra of both the formulations was investigated. **a** SWCNTs-Ag; **b** TP359; **c** FSWCNTs-Ag; **d** SWCNTs-Ag-M. The characteristic peaks for amine groups (N–H) at 1580–1650 cm^−1^, alkane group (C–H) at 3100 cm^−1^, carbonyl group (C=O) at 1760 cm^−1^ and carboxylic group (O–H) at 3300 cm^−1^ was observed on TP359, FSWCNTs-Ag and SWCNTs-Ag-M

**Fig. 3:**
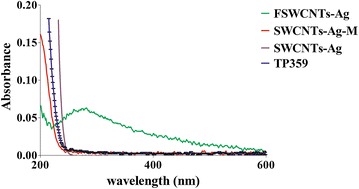
UV–VIS pattern of silver coated single walled carbon nanotubes (SWCNTs-Ag) functionalized with the antimicrobial peptide TP359. SWCNTs-Ag were functionalized with TP359 (FSWCNTs-Ag) or simply mixed with TP359 (SWCNTs-Ag-M) and UV-VIS spectra of both the formulations was investigated and compared with the plan SWCNTs-Ag and TP359. The characteristic peak at ~250 nm on FSWCNTs-Ag ^1^ was observed on TP359 compared to SWCNTs-Ag and SWCNTs-Ag-M

Figure 2 shows the FT-IR pattern of the plain SWCNTs-Ag, TP359, FSWCNTs-Ag (covalent) and SWCNTs-Ag-M (non-covalent). FSWCNTs-Ag showed presence of functional groups for alcohols and phenols (–O–H) at 3400–3600 cm^−1^, carbonyl (–C=O) at 1760 cm^−1^ and amine (N–H) stretches at 1580 cm^−1^ similar to that of TP359, whereas SWCNTs-Ag did not exhibit any of these peaks (Fig. 2a–c). Conversely, the non-covalent bonding of SWCNTs-Ag and the peptide (SWCNTs-Ag-M) showed the presence of alkane (–C–H) at 3000 cm^−1^ and amine stretch at 1580 cm^−1^ similar to that of TP359 however the –O–H stretch was not apparent (Fig. 2d). The FT-IR spectra thus showed the non-covalent and covalent functionalization of SWCNTs-Ag with TP359, later being more apparent (Fig. 2). Similarly, the UV–VIS pattern of FSWCNTs-Ag, SWCNTs-Ag and SWCNTs-Ag-M showed a characteristic peak only on FSWCNTs-Ag at ~250 nm validating the functionalization (Fig. 3).

**Fig. 4:**
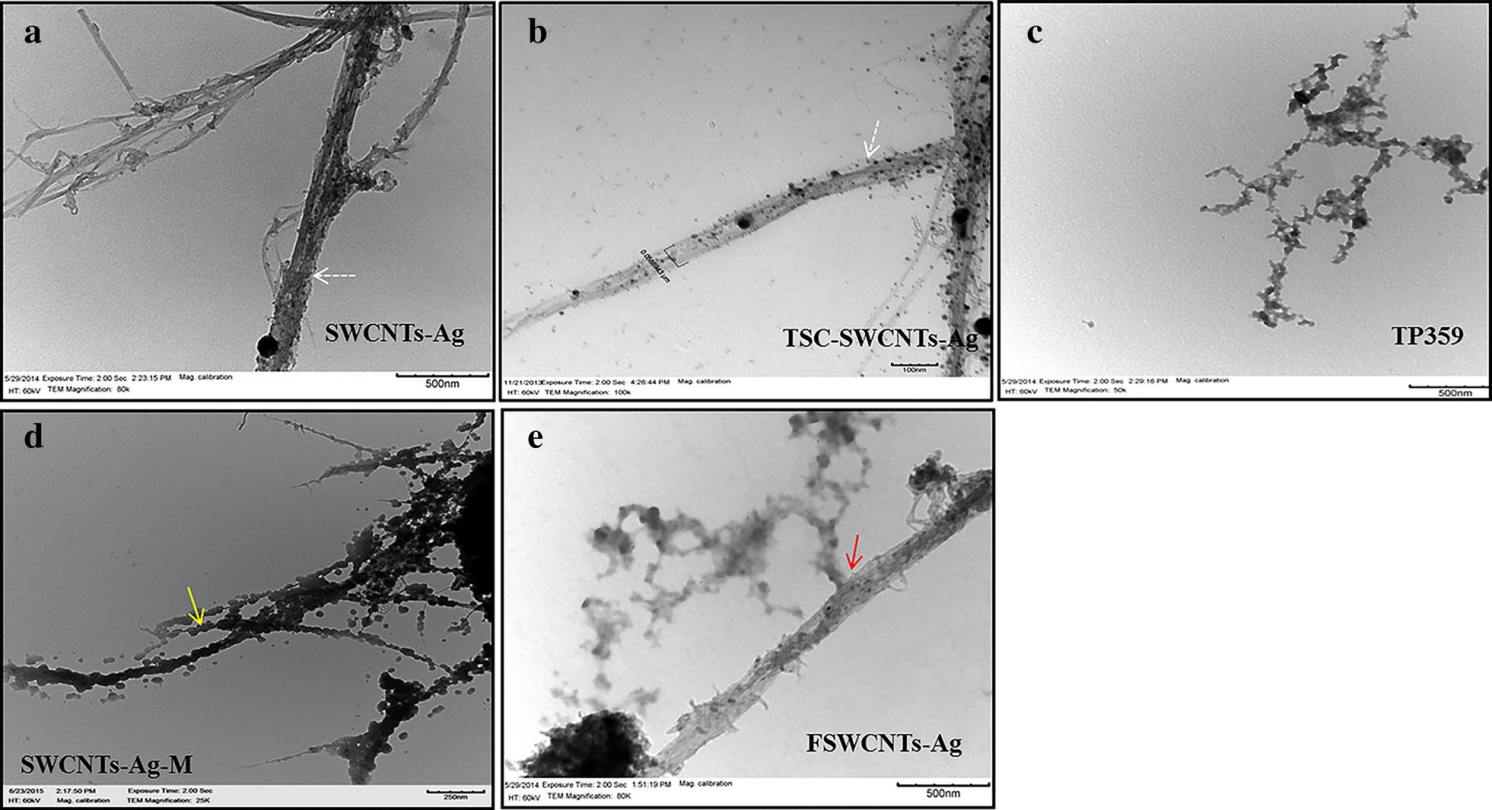
Transmission electron microscopy of silver coated single walled carbon nanotubes (SWCNTs-Ag) functionalized with the antimicrobial peptide TP359. Carboxylated SWCNTs-Ag were functionalized with TP359 and the functionalization of the peptide was confirmed by TEM imaging. **a** SWCNTs-Ag; **b** TSC-SWCNTs-Ag; **c** TP359; **d** SWCNTs-Ag-M; **e** FSWCNTs-Ag. The attachment of the peptide to the SWCNTs-Ag is seen clearly

**Fig. 5:**
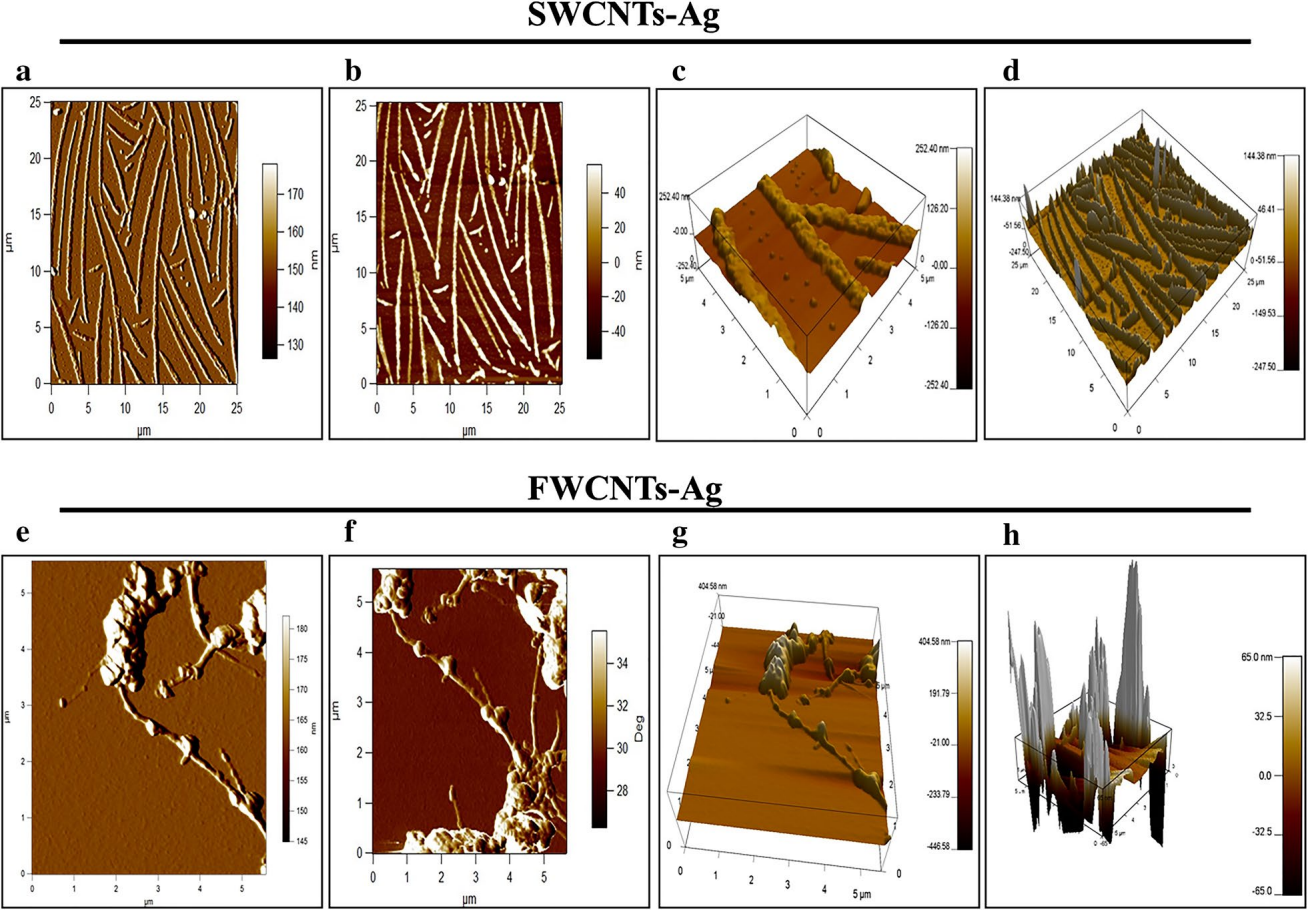
Atomic force microscopy (AFM). Structural characterization of FSWCNTs-Ag and SWCNTs-Ag was carried out to further validate the conjugation of TP359 to SWCNTs-Ag in FSWCNTs-Ag. **a**–**d** AFM analysis of plain SWCNTs-Ag. **e**–**h** AFM analysis of FSWCNTs-Ag. SWCNTs-Ag in two (**a**, **b**) and three (**c**, **d**) dimensional views. FSWCNTs-Ag are presented as two (**e**, **f**) and three (**g**, **h**) dimensional images. The cantilever oscillation frequency was tuned to the resonance frequency of approximately 256 kHz. The set point voltage was adjusted for optimum image quality. Both height and phase information were recorded at a scan rate of 0.7 Hz, and in 512 × 512 pixel format

Further, TEM imaging of SWCNTs-Ag, TSC-SWCNTs-Ag, TP359, SWCNTs-Ag-M and FSWCNTs-Ag was performed and the electronmicrographs are presented in Fig. 4a–e. The silver coating on SWCNTs-Ag and TSC-SWCNTs-Ag can be visualized clearly (Fig. 4a, b, indicated by white dotted arrows) indicating that TSC treatment did not alter the silver coating on the SWCNTs. TEM images of SWCNTs-Ag-M showed that the peptide adhered onto the surface of the SWCNTs-Ag resulting in the coating of SWCNTs-Ag surface with the peptide (Fig. 4d, indicated by a yellow solid arrow). While in the FSWCNTs-Ag samples, the peptide appeared to be attached to SWCNTs-Ag (Fig. 4e, indicated by red solid arrow). Similarly, AFM analysis of FSWCNTs-Ag and plain SWCNTs-Ag was performed to further validate the above findings. Figure 5 shows the surface characteristics of plain SWCNTs-Ag and FSWCNTs-Ag. SWCNTs-Ag appears to be homogeneously distributed on the surface and there was no significant elevation observed on their surface (Fig. 5a–d). In contrast, peptide attachment appears to be evident on FSWCNTs-Ag characterized by significant elevation on the surface (Fig. 5e–h).

### Antibacterial activity of FSWCNTs-Ag compared to SWCNTs-Ag-M, SWCNTs-Ag, TP359 and TSC-SWCNTs-Ag

**Fig. 6:**
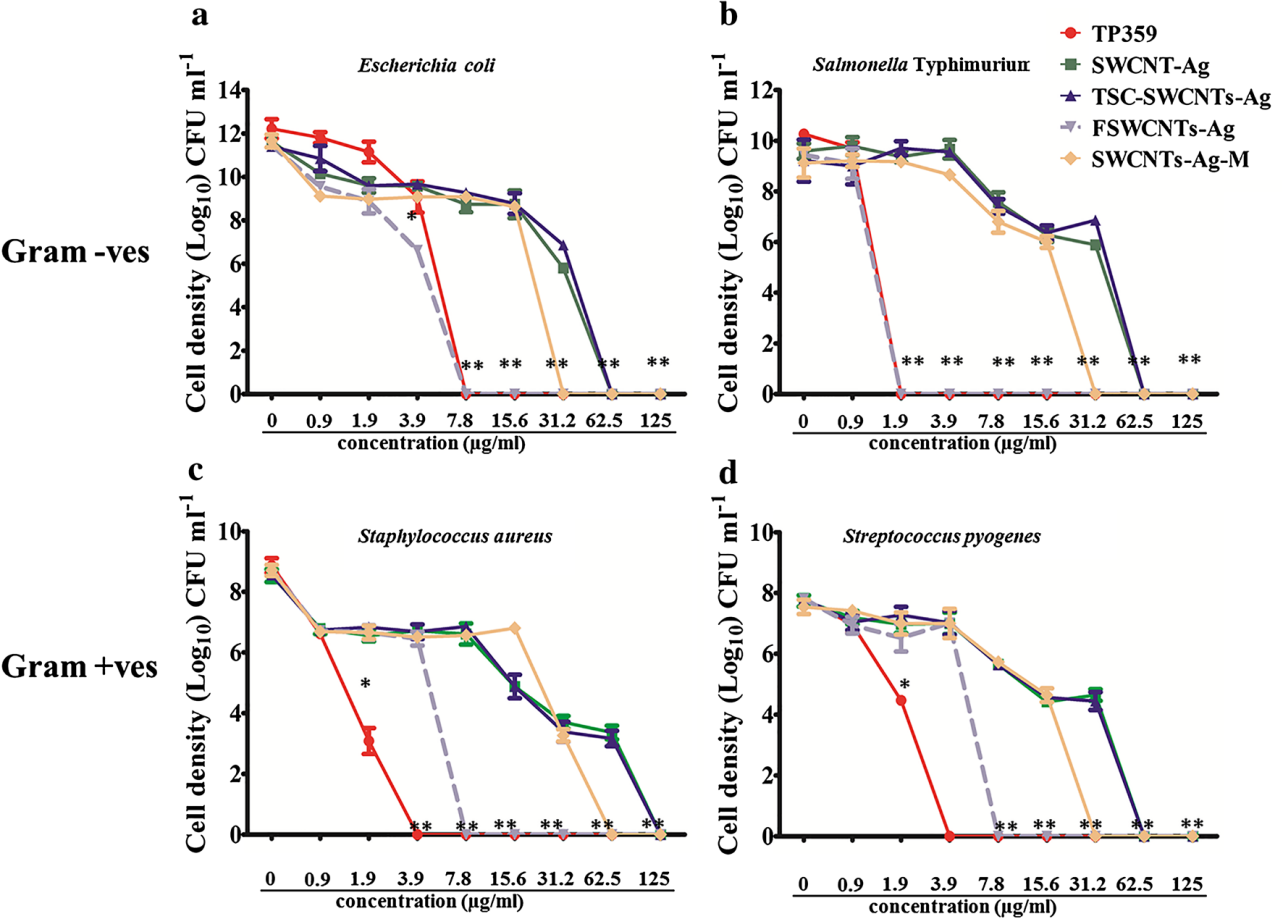
Quantitative analysis of gram positive and gram negative bacteria exposed to various concentrations of nanocomposites. **a** log cfu/ml of surviving *Escherichia coli* upon exposure to peptide and nanocomposites. **b** Quantification of *Salmonella* Typhimurium upon exposure to peptide and nanocomposites. **c** *Staphylococcus aureus* exposed to various nanocomposites. **d** Quantitative analysis of *Streptococcus pyogenes.* Bacteria were grown in LB broth containing various concentrations of nanocomposites and all the cultures were incubated at 37 °C with shaking at 250 rpm and the cfu/ml counts were done at 24 h. Statistical differences were indicates as * when p ≤ 0.05, or ** when value was p ≤ 0.01. *Error bars* represent standard deviations determined from at least four replicates

The MICs of FSWCNTs-AG, SWCNTs-Ag-M, SWCNTs-Ag, TP359 and TSC-SWCNTs-Ag were investigated against two gram negative and two gram positive bacteria. Also the quantitative (cfu/ml) analysis of bacteria exposed to these nanocomposites was determines using plate counting assay. The MICs of FSWCNTs-AG, SWCNTs-Ag-M, SWCNTs-Ag, TP359 and TSC-SWCNTs-Ag are shown in Additional file 1: Figure S1. Table 1 represents the MICs, bactericidal and bacteriostatic concentrations. The MICs for TP359 and FSWCNTs-Ag were lower compared to SWCNTs-Ag, SWCNTs-Ag-M and TSC-SWCNTs-Ag (Additional file 1: Figure S1; Table 1). TP359 exhibited strong antibacterial activity against all four pathogens and the MIC values were in between 7.8–3.9 µg/ml (*Escherichia coli*); 1.9–0.9 µg/ml (*Salmonella* Typhimurium) and 3.9–1.9 µg/ml (for *Staphylococcus aureus* and *Streptococcus pyogenes*). On the other hand, the MIC for SWCNTs-Ag was in between 62.5–31.3 µg/ml and 125–62.5 µg/ml against gram −ve (*Escherichia coli* and *Salmonella* Typhimurium) and gram +ve (*Staphylococcus aureus* and *Streptococcus pyogenes*) bacteria, respectively (Table 1). Compared to SWCNTs-Ag, FSWCNTs-Ag exhibited stronger antibacterial activity at much lower concentration such as 7.8–3.9 µg/ml (against *Escherichia coli*, *Staphylococcus aureus* and *Streptococcus pyogenes*, TP359 concentration is 0.3 µg/ml) and 1.9–0.9 µg/ml (*Salmonella* Typhimurium, TP359 concentration is 0.08 µg/ml). On the contrary for SWCNTs-Ag-M (non-covalent functionalization strategy), the MIC values were still greater than FSWCNTs-Ag and TP359 against *Escherichia coli* and *Salmonella* Typhimurium (31.3–15.6 µg/ml); *Staphylococcus aureus* (62.5–31.3 µg/ml) and *Streptococcus pyogenes* (125–62.5 µg/ml). MIC values of TSC-SWCNTs-Ag against all four bacterial pathogens were similar to that of MICs of SWCNTs-Ag (Additional file 1: Figure S1; Table 1). Additionally, the quantitative analysis of bacteria exposed to these concentrations showed logarithmic decrease in all four bacterial pathogens (Fig. 6a–d). The half maximal inhibitory concentrations (IC50) values (based on the quantitative bacterial growth vs concentrations) for FSWCNTs-Ag (range for all four 1.3–5 µg/ml) were ~tenfolds lower than plain SWCNTs-Ag (range for all four 23–35 µg/ml) (Additional file 1: Table S1). Further, the KB assay results, presented as Table 2 [(*Escherichia coli* and *Salmonella* Typhimurium, Additional file 1: Figure S2) and Additional file 1: Figure S3 (*Staphylococcus aureus* and *Streptococcus pyogenes*)], confirmed the antibacterial activity of FSWCNTs-Ag and TP359 compared to SWCNTs-Ag. The zone of inhibition for TP359 and FSWCNTs-Ag against *E coli* was observed at 20, 10 and 5 µg/ml concentrations whereas SWCNTs-Ag showed slight zone of inhibition only at 20 µg/ml (Table 2, Additional file 1: Figure S2a–c). When *Salmonella* Typhimurium were treated with TP359 and FSWCNTs-Ag at different concentrations, a clear zone of inhibition was observed for all the concentrations compared to SWCNTs-Ag (Table 2; Additional file 1: Figure S2a–c). Similarly, TP359 and FSWCNTs-Ag inhibited the growth of gram positive bacteria at 20, 10 and 5 µg/ml and showed clear zone of inhibition whereas SWCNTs-Ag did not show inhibition at any concentrations (Table 2; Additional file 1: Figure S3a–c).

**Table 1:** MICs, bactericidal and bacteriostatic concentrations

Material	Concentration (µg/ml)			
	*Escherichia coli*	*Salmonella* Typhimurium	*Staphylococcus aureus*	*Streptococcus pyogenes*
TP359				
MICs	7.8–3.9	1.9–0.9	3.9–1.9	3.9–1.9
Bactericidal	7.8*	1.9*	3.9*	3.9*
Bacteriostatic	3.9*	0.9*	1.9*	1.9*
SWCNTs-Ag				
MICs	62.5–31.2	62.5–31.2	125–62.5	125–62.5
Bactericidal	62.5	62.5	125	125
Bacteriostatic	31.2	31.2	62.5	62.5
TSC-SWCNTs-Ag				
MICs	62.5–31.2	62.5–31.2	125–62.5	125–62.5
Bactericidal	62.5	62.5	125	125
Bacteriostatic	31.2	31.2	62.5	62.5
FSWCNTs-Ag				
MICs	7.8–3.9**	1.9–0.9**	7.8–3.9*	7.8–3.9**
Bactericidal	7.8*	1.9**	7.8**	7.8**
Bacteriostatic	3.9	0.9	3.9	3.9
SWCNTs-Ag-M				
MICs	31.2–15.6	31.2–15.6	62.5–31.2	125–62.5
Bactericidal	31.2	31.2	62.5	125
Bacteriostatic	15.6	15.6	31.2	62.5

The p ≤ 0.05 indicating significant * differences, or p ≤ 0.01 indicating highly significant ** differences

**Table 2:** Diameters of zones of inhibition for four bacterial pathogens measured by the Kirby–Bauer disc diffusion assay

Bacteria	Zones of inhibition (mm)												
	Concentration (µg)												
	TP359 (n = 3)				FSWCNTs-Ag (n = 3)				SWCNTs-Ag (n = 3)				Amoxicillin-clavulanic acid (n = 3)
	20	10	5	2.5	20	10	5	2.5	20	10	5	2.5	
*Salmonella* Typhimurium	12 ± 1.5**	10.0 ± 1.0*	6.0 ± 2.0*	5.0 ± 2.0	11.6 ± 2.0*	11.3 ± 1.2**	8.3 ± 1.5*	7.3 ± 1.5*	0.1 ± 0.1	0.13 ± 0.04	0.0 ± 0.0	0.0 ± 0.0	22.0 ± 0.5
*E. coli*	10.0 ± 1.0**	7.0 ± 1.0*	6.6 ± 1.0*	0.0 ± 0.0	10.3 ± 1.5**	7.6 ± 1.5*	6.0 ± 1.7*	0.0 ± 0.0	0.27 ± 1.06	0.15 ± 1.0	0.0 ± 0.0	0.0 ± 0.0	18.0 ± 1.5
*Streptococcus pyogenes*	10.0 ± 1.0**	7.3 ± 1.5*	4.6 ± 0.6*	0.0 ± 0.0	11.0 ± 1.0**	8.3 ± 0.5*	7.0 ± 10*	0.0 ± 0.0	0.12 ± 0.1	0.13 ± 0.15	0.0 ± 0.0	0.0 ± 0.0	18.6 ± 1.5
*Staphylococcus aureus*	10.0 ± 1.0**	7.0 ± 1.0*	5.0 ± 1.0*	0.0 ± 0.0	11.0 ± 1.0**	9.6 ± 2.1*	5.6 ± 1.5*	0.0 ± 0.0	0.1 ± 0.1	0.03 ± 0.05	0.0 ± 0.0	0.0 ± 0.0	17.3 ± 0.5

The p ≤ 0.05 indicating significant * differences, or p ≤ 0.01 indicating highly significant ** differences

### In vitro cell toxicity of TP359, SWCNTs-Ag and FSWCNTs-Ag

**Fig. 7:**
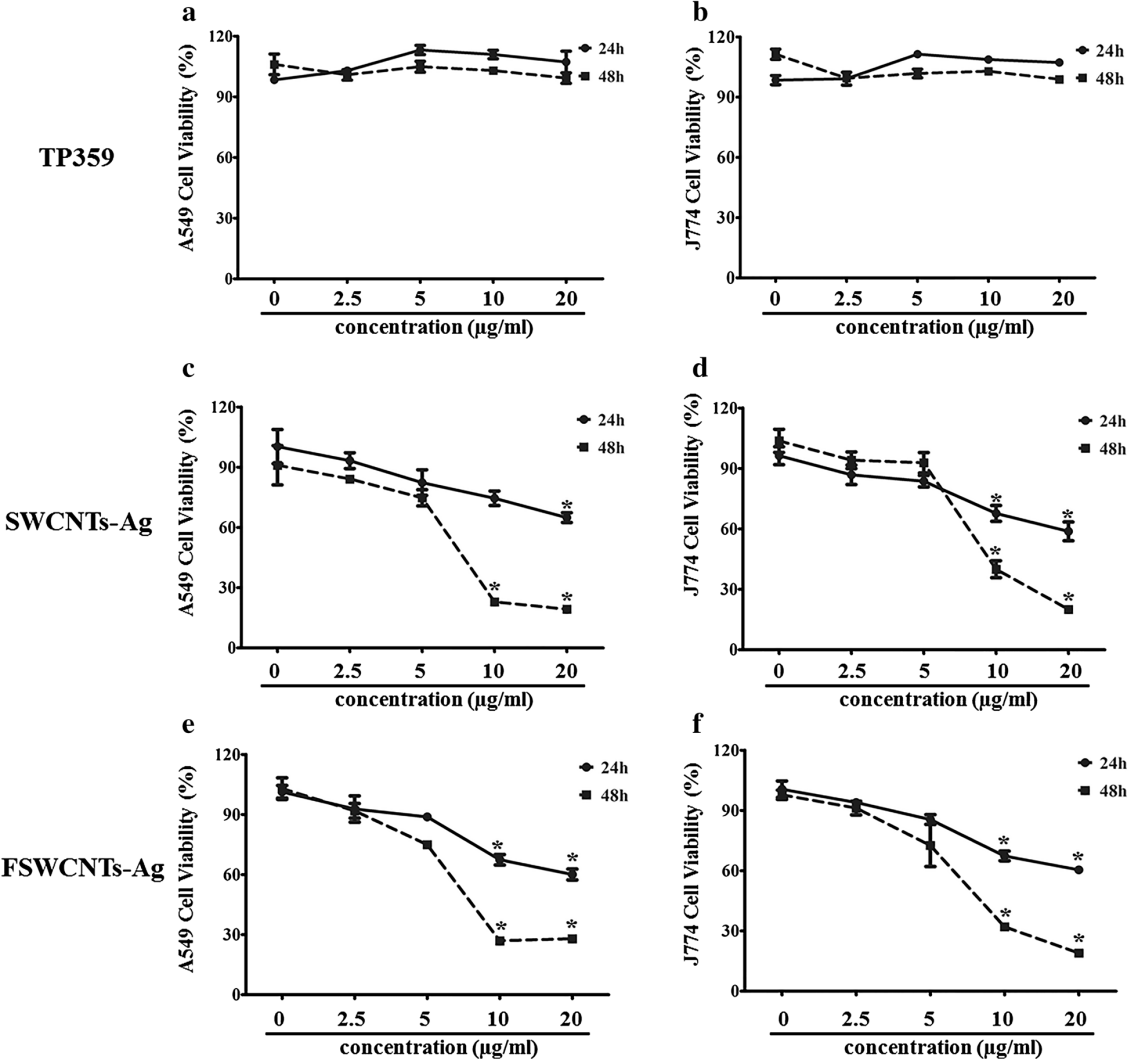
In vitro cytotoxicity assay for TP359, SWCNTs-Ag and FSWCNTs-Ag. **a** Cytotoxicity of A549 cells exposed to TP359. **b** Toxicity to J774 cells exposed to various concentrations of TP359. **c** A549 cell viability exposed to SWCNTs-Ag. **d** Cell viability of J774 treated with SWCNTs-Ag. **e** A549 cells viability treated with FSWCNTsAg. **f** Toxicity of J774 cells exposed to FSWCNTs-Ag. All cells were treated with the peptide and nanocomposites for 24 and 48 h and statistical differences were calculated compared to controls. All values were expressed as fold change expressed compared to non-treated bacteria. * when p ≤ 0.05 indicate significant differences; ** when p ≤ 0.01 indicate highly significant differences. *Error bars* represent standard deviations of the results determined with at least three biological replicates

Next, we explored the cell toxicity of TP359, FSWCNTs-Ag and SWCNTs-Ag at four different concentrations. Figure 7a–f represents cell toxicity of TP359, SWCNTs-Ag and FSWCNTs-Ag in A549 (Fig. 7a, c, e) and J774 cells (Fig. 7b, d, f). TP359 was not toxic to A549 cells or J774 cells at any concentration tested (Fig. 7a, b). On the other hand, FSWCNTs-Ag and SWCNTs-Ag were significantly toxic at 20 and 10 µg/ml (with approximately 30 % cell staining) and were relatively non-toxic at 5 and 2.5 µg/ml concentrations and almost 90 % cells relative to control (Fig. 7c–f).

## Discussion

The present study reports a novel strategy for covalent attachment of antimicrobial peptide to SWCNTs-Ag and antibacterial activity of covalently functionalized SWCNTs-Ag compared to non-covalently functionalized SWCNTs-Ag. In this approach, SWCNTs-Ag were successfully carboxylated using Tri sodium citrate (TSC) and did not involve the use of extreme treatments of SWCNTs-Ag for carboxylation. TSC is a well-known reducing agent and have been engaged in the size controlled synthesis of gold nanoparticles (GNPs) [39, 40]. Besides its role in synthesis of nanoparticles, TSC have also been reported to carboxylate nanoparticles such as GNPs and superparamagnetic iron oxide nanoparticles [41–43]. Congruent with these previous findings, our data showed that treatment of SWCNTs-Ag with TSC resulted in carboxylation of SWCNTs-Ag. However, the striking difference between our study and the previously reported findings is that we used a range of relatively less concentrations of TSC [approximately 0.15 (1:20 ratio) to 0.60 mM (1:5 ratio)] for carboxylation of SWCNTs-Ag compared to 40 mM of TSC that was used to carboxylate the gold nanoparticles [43]. As reported previously [42, 43], the nanoparticles were treated with TSC at high temperatures (90 °C) whereas in this study SWCNTs-Ag were treated with TSC at 37 °C. Carboxylation of SWCNTs generally involves harsh acidic treatments such as combination of H_2_SO_4_ and HNO_3_ at extreme temperatures that has been associated with physical damage to carbon nanotubes [36, 44, 45]. Our results demonstrated that SWCNTs-Ag can be carboxylated without exposure to extreme temperatures or high concentration of chemicals that are associated with degradation.

Further, the carboxylated SWCNTs-Ag were covalently functionalized with the antimicrobial peptide TP359, and non-covalent functionalization was achieved by simply mixing TP359 and plain SWCNTs-Ag. Non-covalent functionalization (SWCNTs-Ag-M) showed adherence of the peptide onto the surface of the SWCNTs-Ag whereas covalent functionalization (FSWCNTs-Ag) showed the attachment of the peptide to SWCNTs-Ag. It has been established that covalent functionalization of nanoparticles exhibit greater stability and improved activity [36–38]. In contrast, non-covalent functionalization is highly unstable and less effective than the covalent bonding [33]. The protocol described herein provides a relatively simple and stable approach to the covalent functionalization of the SWCNTs-Ag that neither involves extreme chemical treatment, nor causes damage to the carbon nanotubes due to exposure to high temperatures.

Evaluation of antibacterial activity of FSWCNTs-AG, SWCNTs-Ag-M, SWCNTs-Ag, TP359 and TSC-SWCNTs-Ag revealed that the antibacterial concentrations of FSWCNTs-Ag were much lower compared to SWCNTs-Ag-M and TSC-SWCNTs-Ag, suggesting that the covalent conjugation of TP259 with SWCNTs-Ag showed increased efficacy compared to their counterparts as far as the antibacterial activity was concerned. It is therefore interesting to examine the mechanism of antibacterial action of the FSWCNTs-Ag compared to the peptide alone and SWCNTs-Ag. The present study thus defines a novel protocol to functionalize the silver coated single walled carbon nanotubes with the peptide which can be used to further evaluate the exact mechanism for antibacterial action for peptide functionalized SWCNTs-Ag compared with peptide and plain SWCNTs-Ag alone. The functionalization of SWCNTs with DNA, RNA or chemotherapeutic drugs has shown remarkable success in achieving the desired targets for the action of these biological molecules [30, 32–35]. However, it is known that the SWCNTs exhibit less potent antibacterial activity compared to SWCNTs-Ag (MIC concentrations 62.5 µg/ml). Therefore in this study, we attempted the functionalization of SWCNTs-Ag (vs. un-functionalized SWNCTs) with a known antimicrobial peptide in order to reduce the effective antimicrobial dosage of SWCNTs-Ag. This may has potential to reduce off the toxicity of SWCNTs-Ag to human cells and may reduce the emergence of drug resistance phenotypes. Our results suggested that covalently conjugating the peptide to SWCNTs-Ag effectively reduced the antibacterial dosage of the SWCNTs-Ag as well as TP359. We also demonstrated that noncovalent functionalization did not show any significant antibacterial activity compared to SWCNTS-Ag. It should be noted that FSWCNTs-Ag and SWCNTs-Ag-M, were washed after incubation and resulted in the loss of unbound (in case of FSWCNTs-Ag) and non-adherent (in case of SWCNTs-Ag-M) TP359. The loss of the TP359 in the supernatant of SWCNTs-Ag-M was greater than the supernatant of FSWCNTs-Ag (Table 3). As expected, this suggests that non-covalent binding of the peptide (SWCNTs-Ag-M) is relatively unstable and is easily removed in the supernatant and therefore SWCNTs-Ag-M did not exhibit any significant antibacterial activity as good as FSWCNTs-Ag. Although the supernatant of FSWCNTs-Ag lost most (almost 80 %) of the unbound TP359, FSWCNTs-Ag still exhibited effective antibacterial properties which indicate that the covalent bonding of the peptide to SWCNTs-Ag is stable. Our results also indicated that treatment of SWCNTs-Ag with TSC did not affect the antibacterial activity of SWCNTs-Ag. TSC is used widely as a reducing agent. It is possible that the treatment with TSC may reduce the silver coating on the SWCNTs and could result into the loss of antibacterial activity. However, based on TEM and the antibacterial activity assay we did not observe loss of the silver coating on the SWCNTs-Ag. This may be a result of using lower concentrations of TSC for carboxylation of SWCNTs-Ag. The results from the present study are interesting as the novel FSWCNTs-Ag exhibited excellent antibacterial activity. However, it is important to further evaluate the antibacterial activity in detail using advanced methodology such as scanning electron microscopy, transmission electron microscopy, qRTPCR studies to evaluate gene expression, haemolytic activity etc.

**Table 3:** TP359 peptide concentration measure by BSA assay

Nanocomposite^a^	TP359 concentration (µg/ml)					
	Initial^b^	W_1_^c^	W_2_^d^	W_3_^e^	W_4_^f^	Final [(initial − (W1 + W2 + W3)]^g^
FSWCNTs-Ag	1000	731	48.7	12.7	0	208
SWCNTs-Ag-M	1000	818	76.2	29.7	0	76.1

^a^Silver coated single walled carbon nanotubes (SWCNTs-Ag) were functionalized with TP359 either covalently (FSWCNTs-Ag) or non-covalently (SWCNTs-Ag-M)

^b^Starting concentration of TP359 used for functionalization

^c^First wash after centrifugation

^d^Second wash after centrifugation

^e^Third wash after centrifugation

^f^Fourth wash after centrifugation

^g^Final concentration of TP359 that was present on the functionalized nanocomposites

Finally the cytotoxicity of FSWCNTs-AG, SWCNTs-Ag and TP359 was evaluated. It has been reported that non covalent functionalization of SWCNTs or SWCNTs-Ag using non-toxic polymers such as poly ethylene glycol reduces the toxicity in eukaryotic cells [11, 18, 41, 44, 45]. In our previous report we have shown successfully that non-covalently pegylated SWCNTs-Ag are less toxic compared to non-pegylated SWCNTs-Ag at their bactericidal concentrations [11]. In the present study our results suggest that covalent functionalization with peptide did not reduce the toxicity of SWCNTs-Ag, as SWCNTS-Ag and FSWCNTs-Ag were toxic at higher concentrations (20 and 10 µg/ml) and the toxicity was reduced further at lower concentrations (Fig. 7). However, the important difference between the two formulations is that the FSWCNTs-Ag retained antibacterial activity at lower concentrations whereas non-functionalized SWCNTs-Ag (although being non-toxic at lower concentrations) did not exhibit antibacterial activity at equal concentrations. In particular, MIC concentrations of the FSWCNTs-Ag against *Salmonella* Typhimurium are much lower than its not-toxic concentrations to eukaryotic cells. It is still not clear if the conjugation efficiency can be enhanced by increasing the peptide concentration or how different conditions, such as pH variation, temperature etc., affect the stability of the FSWCNTs-Ag; these factors are under investigation.

## Conclusions

In conclusion, the present study describes a novel approach for covalent bio-conjugation of silver coated SWCNTs with an antimicrobial peptide. Functionalization exhibited an “additive effect” relationship between the SWCNTs-Ag and TP359 as far as antibacterial activity is concerned. This additive effect is evidenced by the requirement of much lower concentrations of the TP359 (0.3–0.08 µg/ml) and SWCNTs-Ag (7.8–1.9 µg/ml) in the FSWCNTs-Ag to obtain the desired antibacterial effect against all bacterial pathogens compared to the plain TP359 (MIC: 7.8–1.9 µg/ml) and SWCNTs-Ag (MIC: 125–62.5 µg/ml). Also the IC50 values of FSWCNTs-Ag (~4–5 µg/ml) were much lower as opposed to plain SWCNTs-Ag IC50: ~31–35 µg/ml) More importantly, our results showed that the FSWCNTs-Ag are non-toxic to murine macrophages and Hep2 cells at their MIC concentrations (5–2.5 µg/ml). The protocol described in the present study provides a beneficial approach to bio-conjugate SWCNTs-Ag and will aid into development of novel and biologically important nanomaterials.

## Methods

### Preparation of nanomaterial and antimicrobial peptide TP359

Silver (typically 40–50 wt%) coated single walled carbon nanotubes (SWCNTs-Ag), were purchased from NanoLab, Inc. Waltham, MA, USA. One milligrams of SWCNTs-Ag were dispersed in 1 ml of sterile distilled water to produce its aqueous dispersion. The dispersion was immediately sonicated for 1–2 h to obtain the SWCNTs-Ag dispersion (1 mg/ml). Similarly, 1 mg of a novel antimicrobial peptide from Therapeutic peptides Inc. (Baton Rouge, LA, USA), TP359 of 5 amino acids, was solubilized in sterile water. TP359 is a lipidated cationic oligopeptide (LCOP), myristoyl-KKALK_d_ amide (US Patent 8431523).

### Carboxylation of SWCNTs-Ag using tri-sodium citrate (TSC)

SWCNTs-Ag were carboxylated using Trisodium citrate dihydrate (TSC) (Alfa Aesar, Ward Hill, MA, USA) as described previously with fewer modifications [42, 43, 46]. Briefly, SWCNTs-Ag were mixed with TSC at different ratios of TSC to SWCNTs-Ag (1 mg/ml) such as 1:20 (1 part of TSC to 20 parts of SWCNTs-Ag), 1:10, 1:7.5 and 1:5. The mixture was subjected to continuous stirring at 37 °C for 4 h. The samples were designated as TSC-SWCNTs-Ag, vacuum dried and the coating of carboxyl group on SWCNTs-Ag surface was further analyzed using FT-IR as described elsewhere [11].

### Bio-conjugation of TP359 to carboxylated SWCNTs-Ag using covalent bonding

The carboxylated SWCNTs-Ag were covalently conjugated with TP359 using a 1-Ethyl-3-(3-dimethylaminopropyl) carbodiimide (EDC, Sigma Aldrich, St. Louis, MO, USA) and N-Hydroxysuccinimide (NHS, Sigma Aldrich, St. Louis, MO, USA). Briefly, 1 mg of TSC-treated SWCNTs-Ag (1:20) were mixed with 0.5 mg of EDC and 0.25 mg of NHS and subjected to continuous stirring for 1 h, followed by addition of 1 mg of TP359 and stirring of the mixture for 4 h. Unbound peptide was removed by repeated centrifugation (at least four times) at 40,000×*g* for 50 min and the functionalized SWCNTs-Ag (FSWCNTs-Ag) were finally re-suspended in sterile nuclease-free water 1 ml. Each wash after centrifugation was collected to measure the peptide concentration using BCA™ protein assay kit (Thermo scientific, Rockford, IL, USA). Functionalization was further assessed by using FT-IR spectroscopy, UV–VIS spectra, atomic force microscopy (AFM) and transmission electron microscopy (TEM).

### Non-covalent functionalization of TP359 and SWCNTs-Ag

Non covalent functionalization of SWCNTs-Ag with TP359 was carried out to compare with the covalent functionalization strategy. For this purpose, 1 mg of SWCNTs-Ag were mixed with 1 mg of TP359 and kept for stirring of the mixture for 4 h. The samples were subjected to repeat centrifugation (at least four times) at 40,000×*g* for 50 min and the sample was finally re-suspended in 1 ml of sterile nuclease-free water and denoted as SWCNTs-Ag-M. Each wash after centrifugation was collected to measure the peptide concentration using BCA™ protein assay kit (Thermo scientific, Rockford, IL, USA). The concentration of TP359 in the covalently and non-covalently attached SWCNTs-Ag is presented in Table 3.

### Characterization of TSC-treated SWCNTs-Ag, FSWCNTs-Ag, SWCNTs-Ag-M and TP359 using FT-IR, TEM

The nanocomposite formulations were characterized using FT-IR analysis and TEM imaging to confirm the presence of carboxyl group in TSC-SWCNTs-Ag and covalent attachment of the peptide to SWCNTs-Ag. In order to confirm the carboxylation of SWCNTs-Ag, FTIR spectra of different ratio of TSC to SWCNTs-Ag (such as 1:20, 1:10, 1:7.5 and 1:5) were recorded and compared to only TSC and SWCNTs-Ag. The FT-IR pattern of FSWCNTs-Ag, SWCNTs-Ag-M, TP359 and SWCNTs-Ag were compared to confirm the functionalization. The spectra were recorded in attenuated total reflectance (ATR) mode using an infrared (IR) spectrophotometer (Nicolet 380 FT-IR; Thermo Fisher Scientific) as 64 scans per sample, ranging from 400 to 4000 cm^−1^ and a resolution of 4 cm^−1^. The sample chamber was purged with dry N_2_ gas.

TEM (TEM, Zeiss EM 10C 10CR, Carl Zeiss Meditec, Oberkochen, Germany) was used to examine the covalent attachment of TP359 to SWCNTs-Ag and compared with TSC-SWCNTs-Ag, SWCNTs-Ag-M, TP359 and SWCNTs-Ag. The samples for TEM were prepared as described previously [12]. Briefly, the samples were sonicated, diluted ten times and placed on the carbon-coated copper grid (200 mesh) and observed by TEM.

### AFM

Approximately 50 μl of SWCNTs-Ag and FSWCNTs-Ag solution were dropped on a fresh cleaved glass slide and air-dried for overnight under clean bench. The dried samples were used to conduct atomic force microscopy (AFM; Asylum MF3D Conductive AFM, Santa Barbara, California). Air contact mode and standard silicon cantilevers (Budget Sensors, Sofia, Bulgaria) 300 μm in length and 30 μm in width were used for imaging. The cantilever oscillation frequency was tuned to the resonance frequency of approximately 256 kHz. The set point voltage was adjusted for optimum image quality. Both height and phase information were recorded at a scan rate of 0.7 Hz, and in 512 × 512 pixel format. AFM images in a large scanning area were processed using Asylum software (Asylum Research).

### Ultraviolet visualization (UV–Vis)

UV–Vis was employed to ascertain the functionalization of the peptide TP359 on SWCNTs-Ag. SWCNTs-Ag, FSWCNTs-Ag and SWCNTs-Ag-M nanoparticles were each diluted in deionized water and their absorbance and spectral wavelengths were assessed using the DU 800UV/Vis spectrophotometer (Beckman Coulter, Fullerton, CA).

### Bacterial experiments

*Salmonella enterica* serovar Typhimurium (ATCC^®^ 13311™), *Escherichia coli* (ATCC^®^ 25922™), *Staphylococcus aureus* (ATCC^®^ 9144™) and *Streptococcus pyogenes* (ATCC^®^ 8135™) were purchased from American Type Culture Collection (ATCC, VA USA). Bacteria were grown at 37 °C in Luria–Bertani (LB) broth (Difco, Sparks, MD, USA) with continuous shaking until the optical density (OD) was 0.6–0.8 (at 600 nm). Bactericidal activity of SWCNTs-Ag, TP359, TSC-SWCNTs-Ag, FSWCNTs-Ag and SWCNTs-Ag-M was investigated using the parameters such as minimum inhibitory concentrations (MIC), quantitative growth analysis and the Kirby-Bauer (KB) disc diffusion assay against all four pathogens.

### Determination of minimum inhibitory concentration (MIC)

The MIC values for SWCNTs-Ag, TP359, TSC-SWCNTs-Ag, FSWCNTs-Ag and SWCNTs-Ag-M were evaluated in quadruplet wells of sterile 96-well microtiter plates using the broth microdilution assay as previously described broth microdilution procedure [47, 48]. Briefly, 1 × 10^5^ cfu/ml of the bacteria were exposed to doubling concentrations of the samples starting at 0.9 μg/ml. For this purpose, the optical density of bacterial culture was measured at 600 nm and the cfu/ml was determined using standard curve equation. One milliliter of media containing 1 × 10^9^ cfu/ml of the bacteria were diluted tenfolds (10^8^, 10^7^, 10^6^, 10^5^) subsequently to obtain 1 × 10^5^ cfu/ml of the bacteria. All plates were sealed lightly (with ventilation) and then incubated at 37 °C for 24 h. Each plate consisted of eight dilutions of the samples, one negative control (no sample or no bacterial culture) and one positive control (only bacterial culture without samples). The concentration of the first well with no turbidity was considered as the MIC. The inhibition of bacterial growth was determined by measuring absorbance at 600 nm with a TECAN Sunrise™ enzyme-linked immunosorbent assay (ELISA) plate reader (Tecan US, Inc Morrisville, NC, USA). To avoid the background absorbance of the nanoparticles, the plates were read at 0 h before keeping in the incubator. Then the 0 h readings were subtracted from the 24 h readings to obtain the actual absorbance values at OD600. All experiments were repeated at least three times. To determine whether there is a synergistic or additive effect between the TP359 and SWCNTs-Ag in the FSWCNTs-Ag nanocomposite, we determined the fractional inhibitory concentration (FIC) test as described earlier [49]. The combined antibacterial effect of nanoparticles A and B (where A is TP359, B is SWCNTs-Ag, and AB is FSWCNTs-Ag) was calculated using the following formula as:

$${\text{FIC index}} = \left[ {{{{\text{MIC}}\left( {\text{AB}} \right)} \mathord{\left/ {\vphantom {{{\text{MIC}}\left( {\text{AB}} \right)} {{\text{MIC}}\left( {\text{A}} \right)}}} \right. \kern-0pt} {{\text{MIC}}\left( {\text{A}} \right)}}} \right] + \left[ {{{{\text{MIC}}\left( {\text{AB}} \right)} \mathord{\left/ {\vphantom {{{\text{MIC}}\left( {\text{AB}} \right)} {{\text{MIC}}\left( {\text{B}} \right)}}} \right. \kern-0pt} {{\text{MIC}}\left( {\text{B}} \right)}}} \right].$$

The results were indicated as: FIC index values below 0.5 indicate synergistic effect, above 2 indicate antagonistic effects and values between 0.5 and 2.0 indicate additive effects.

### Quantitative growth analysis of bacteria

Growth of all four pathogens was quantified at 24 h post-exposure to SWCNTs-Ag, TP359, TSC-SWCNTs-Ag, FSWCNTs-Ag and SWCNTs-Ag-M. 1 × 10^5^ cfu/ml of the bacteria were exposed to doubling concentrations of the samples starting at 0.9 μg/ml. The cultures were incubated at 37 °C with shaking at 250 rpm for 24 h. Post incubation, 1 ml aliquots of bacterial culture were collected, subjected to serial tenfold dilution in sterile LB broth, and appropriate dilution was then spread on PCA to determine the cfu/ml. Each sample was analyzed in quadruplet. The bacterial cfu/ml for each sample was counted and the logarithmic decrease in bacterial growth was expressed as log_10_ cfu/ml value of each sample. The IC50 values for plain SWCNTs-Ag and FSWCNTs-Ag were calculated using % inhibition on Y axis and concentrations on X axis.

### KB assay

Based on the MIC findings, the antibacterial activity of TP359 and FSWCNTs-Ag at four concentrations such as 20 (4× of MIC), 10 (2× of MIC), 5 (MIC), 2.5 (0.5× of MIC) μg/ml was further tested using the KB assay against all four pathogens as described earlier with fewer modifications and was compared with similar concentrations of SWCNTs-Ag [50]. Bacterial suspensions of each bacterial strain (10^5^ cfu/ml) were swabbed on the surface of Mueller–Hinton agar plates and filter paper discs (Fisher Scientific, MO) containing different concentrations of SWCNTs-Ag, TP359 and FSWCNTs-Ag, were placed on the plate. A broad spectrum combination of amoxicillin and clavulanic acid (30 μg) was used as a positive control (BD, BBL™, USA). Plates were incubated at 37 °C overnight and ‘zones of inhibition’ were observed.

### In vitro cell toxicity assay

The cell toxicity to SWCNTs-Ag, TP359 and FSWCNTs-Ag was determined using Cell Titer 96^®^ Non-Radioactive cell proliferation kit (Promega, Madison, WI). A549 (human lung carcinoma) and J774 (murine macrophages) cell lines were used for the cytotoxicity assay. Cytotoxicity was determined using a colorimetric assay based on the reduction of tetrazolium dye MTT [3-(4,5-dimethylthiazol-2yl)-2,5-diphenyltetrazolium bromide). As per the manufacturer’s protocol, 1 × 10^4^ cells/well in 100 μl of minimum essential medium-10 (MEM-10, Gibco, Life technologies, Grand Island, NY) were seeded into a 96-well plate. After overnight incubation at 37 °C in 5 % CO_2_ humidified atmosphere, the media from the 96-well plate were replaced with the MEM-10 containing 20, 10, 5 and 2.5 μg/ml of SWCNTs-Ag, TP359 and FSWCNTs-Ag. The treated cells were further incubated at 37 °C and 5 % CO2 for 24 and 48 h. At the end of the corresponding incubation time, 15 μl of MTT dye was added into the each well, the plates were sealed using aluminum foil and was allowed to incubate again for the next 4 h. The reaction was then stopped with 100 μl of stop solution. The absorbance of the plate was measured at 570 nm on a TECAN Sunrise™ enzyme-linked immunosorbent assay (ELISA) plate reader (Tecan US, Inc., Morrisville, NC, USA). Non-treated cells, in growth media, were used as a control.

### Statistical analyses

All data are expressed as the mean ± standard deviation (SD) unless otherwise specified. Analyses were performed with GraphPad Prism Version 4 software (GraphPad Software, Inc., La Jolla, CA). Statistical differences for MICs and cytotoxicity assay were evaluated by using post hoc pairwise comparison of two-way ANOVA. Differences were considered to be statistically significant when the p values were ≤0.05 or 0.01.

## Additional file

10.1186/s12951-016-0211-z IC50 table, MIC graph and KB assay images against gram-positive and gram-negative bacteria.

## Abbreviations

APs: antimicrobial peptides
ANOVA: analysis of variance
ATR: attenuated total reflectance
ELISA: enzyme-linked immunosorbent assay
FIC: fractional inhibitory concentration
FSWCNTs-Ag: covalently functionalized silver coated single walled carbon nanotubes
FT-IR: fourier transform infrared spectroscopy
IC50: the half maximal inhibitory concentration
IR: infrared
MIC: minimum inhibitory concentrations
MTT: 3-(4,5-dimethylthiazol-2yl)-2,5-diphenyltetrazolium bromide
OD: optical density
SWCNTs: single walled carbon nanotubes
SWCNTs-Ag: silver coated single walled carbon nanotubes
SWCNTS-Ag-M: non-covalently functionalized silver coated single walled carbon nanotubes
TEM: transmission electron microscopy
TP359: therapeutic peptide 359
TSC: tri-sodium citrate
TSC-SWCNTs-Ag: tri-sodium treated silver coated single walled carbon nanotubes
UV-VIS: ultraviolet visualization
