# Supplementary material for: A novel covalent approach to bio-conjugate silver coated single walled carbon nanotubes with antimicrobial peptide
Source: J Nanobiotechnology. 2016 Jul 13;14:58. doi: 10.1186/s12951-016-0211-z (PMC4944237; doi:10.1186/s12951-016-0211-z)
Supplement: Supplementary file 1 — 10.1186/s12951-016-0211-z IC50 table, MIC graph and KB assay images against gram-positive and gram-negative bacteria. [file 12951_2016_211_MOESM1_ESM.docx]

**A novel covalent approach to bio-conjugate silver coated single walled carbon nanotubes with antimicrobial peptide**

Atul A. Chaudhari^1^, Vida Dennis^1^, Shree R. Singh^1^, Don R. Owen^2^, Chris Palazzo^2^, [Robert D Arnold](http://www.ncbi.nlm.nih.gov/pubmed/?term=Arnold%20RD%5Bauth%5D)^3^, Michael E. Miller^4^, Shreekumar R. Pillai^1*^

^1^ Center for Nanobiotechnology Research, Alabama State University, Montgomery, AL, USA

^2^ Therapeutic Peptides Inc., 7053 Revenue Drive, Baton Rouge, LA-70809, USA

^3^ Department of Drug Discovery and Development, Auburn University, AL, USA

^4^ Research Instrumentation Facility, Auburn University, AL, USA

Corresponding Author:

* E-mail: [spillai@alasu.edu](mailto:spillai@alasu.edu)

Table S1. IC50 values for FSWCNTs-Ag as opposed to SWCNTs-Ag

| Material | | concentration (µg/ml) | | | |
| --- | --- | --- | --- | --- | --- |
|  |  | *Escherichia coli* | *Salmonella* Typhimurium | *Staphylococcus aureus* | *Streptococcus pyogenes* |
| SWCNTs-Ag | IC50 | 31.2 | 35.85 | 23.4 | 35.75 |
| FSWCNTs-Ag | IC50 | 4.3^**^ | 1.34^**^ | 4.91^**^ | 5.73^*^ |

**Supplementary information**

**Figures**

**
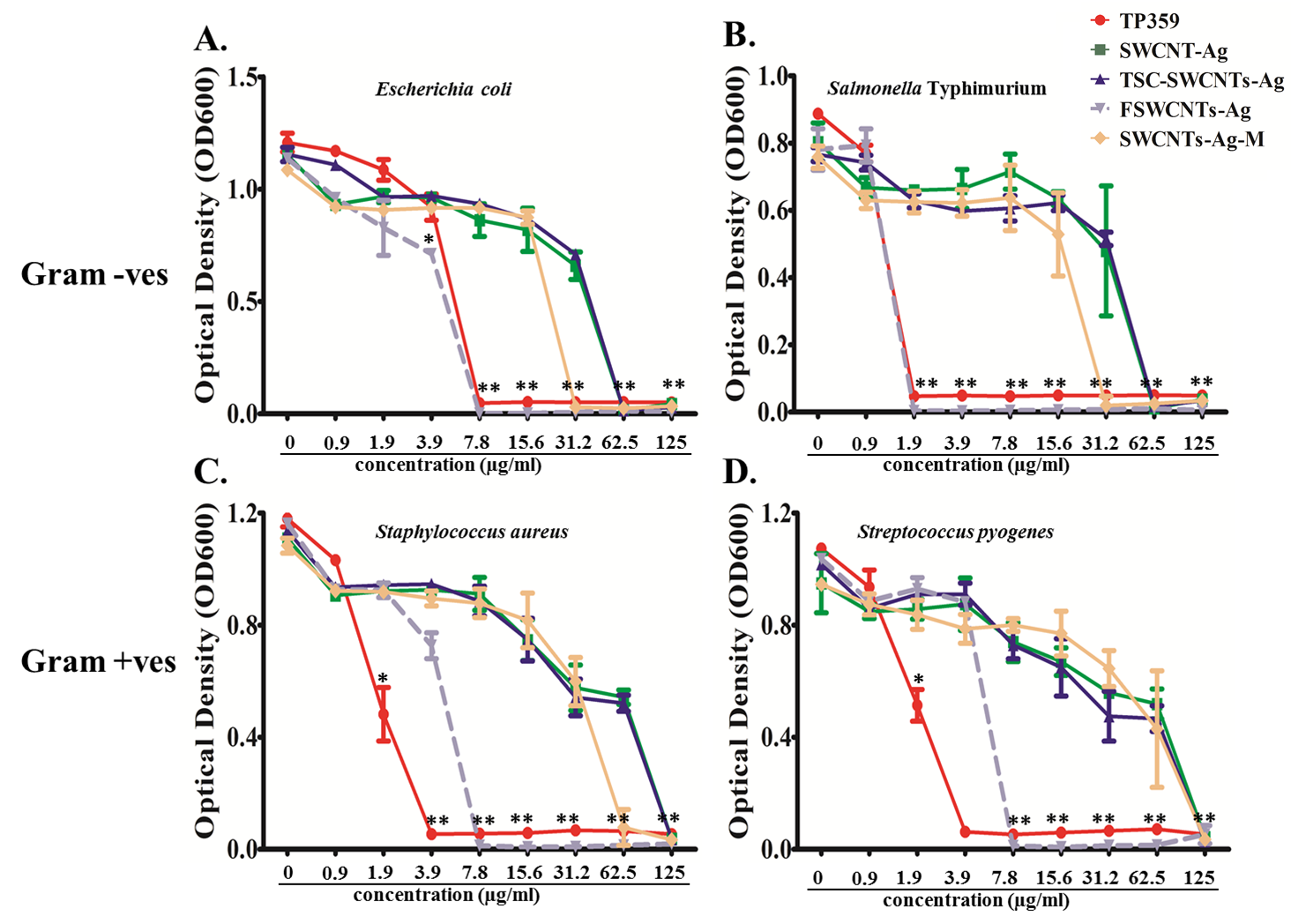
**

**Figure S1. Minimum inhibitory concentrations (MICs) assay gainst gram- negative and gram-positive organisms.** The bacteria (1 x 10^5^ cfu/mL) were exposed to doubling concentrations of SWCNTs-Ag; TP359; TSC-SWCNTs-Ag; FSWCNTs-Ag and SWCNTs-Ag-M. (a) *Escherichia coli*.; (b) *Salmonella* Typhimurium; (c) *Staphylococcus aureus*; (d) *Streptococcus pyogenes*. All the plates were sealed and incubated at 37 °C and the optical density at 600 nm (OD 600) was determined after 24 h. All values were considered to be significant at p ≤ 0.05 or 0.01. ** p ≤ 0.01 indicating highly significant differences. Error bars represent standard deviations determined from at least six replicates.


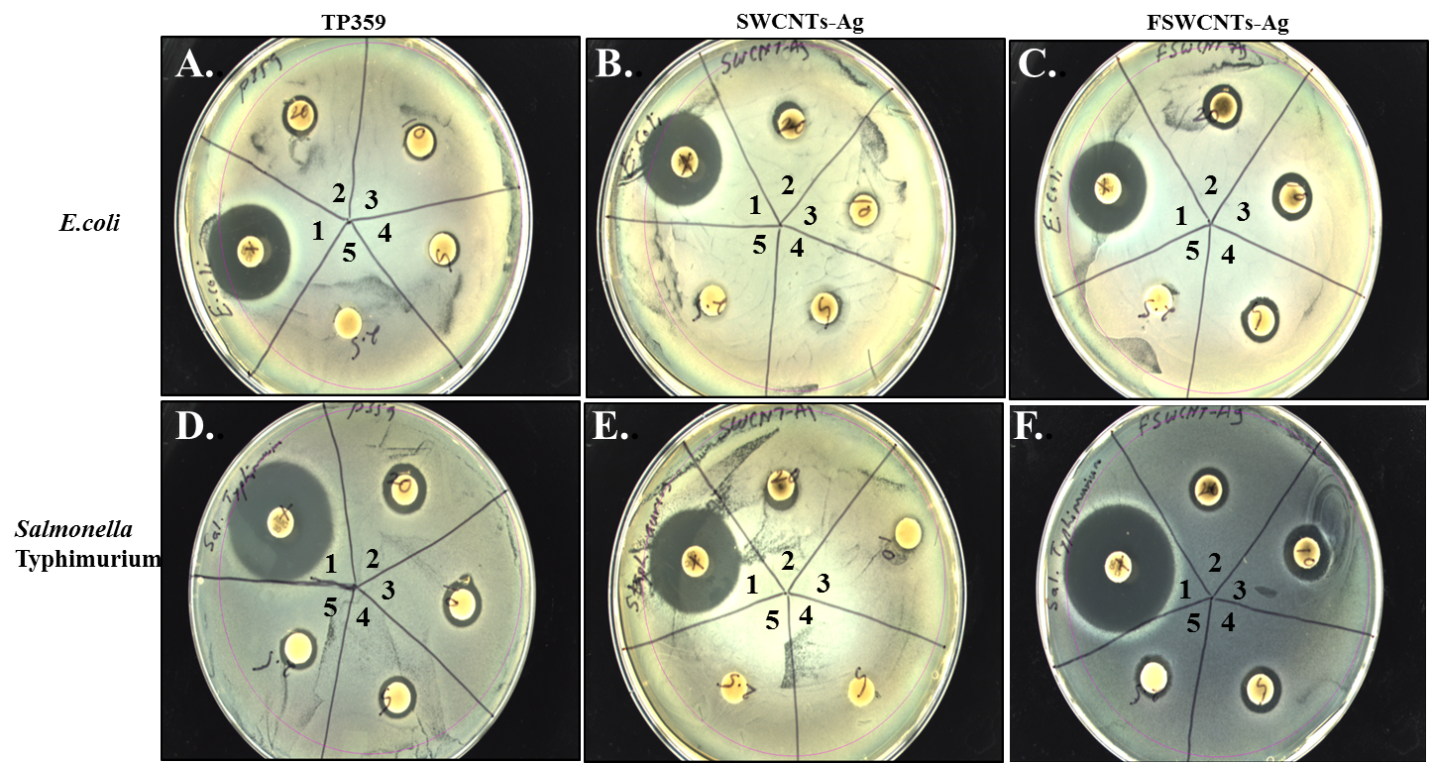


**Figure S2.** **Evaluation of zone of inhibition using Kirby–Bauer disc diffusion assay against gram- negative pathogens.** The zone of inhibition around discs containing MIC concentrations of TP359, SWCNTs-Ag and FSWCNTs-Ag and the broad spectrum antibiotic amoxicillin–clavulanic acid (30 µg) can be observed for both the gram- negative pathogens such as (a-c) *Escherichia coli* and (d-f) *Salmonella* Typhimurium. The numbers indicated represents the concentrations of TP359, SWCNTs-Ag and FSWCNTs-Ag; 1: antibiotic control; 2: 20 µg/mL; 3: 10 µg/mL; 4: 5 µg/mL; 5: 2.5 µg/mL.

**
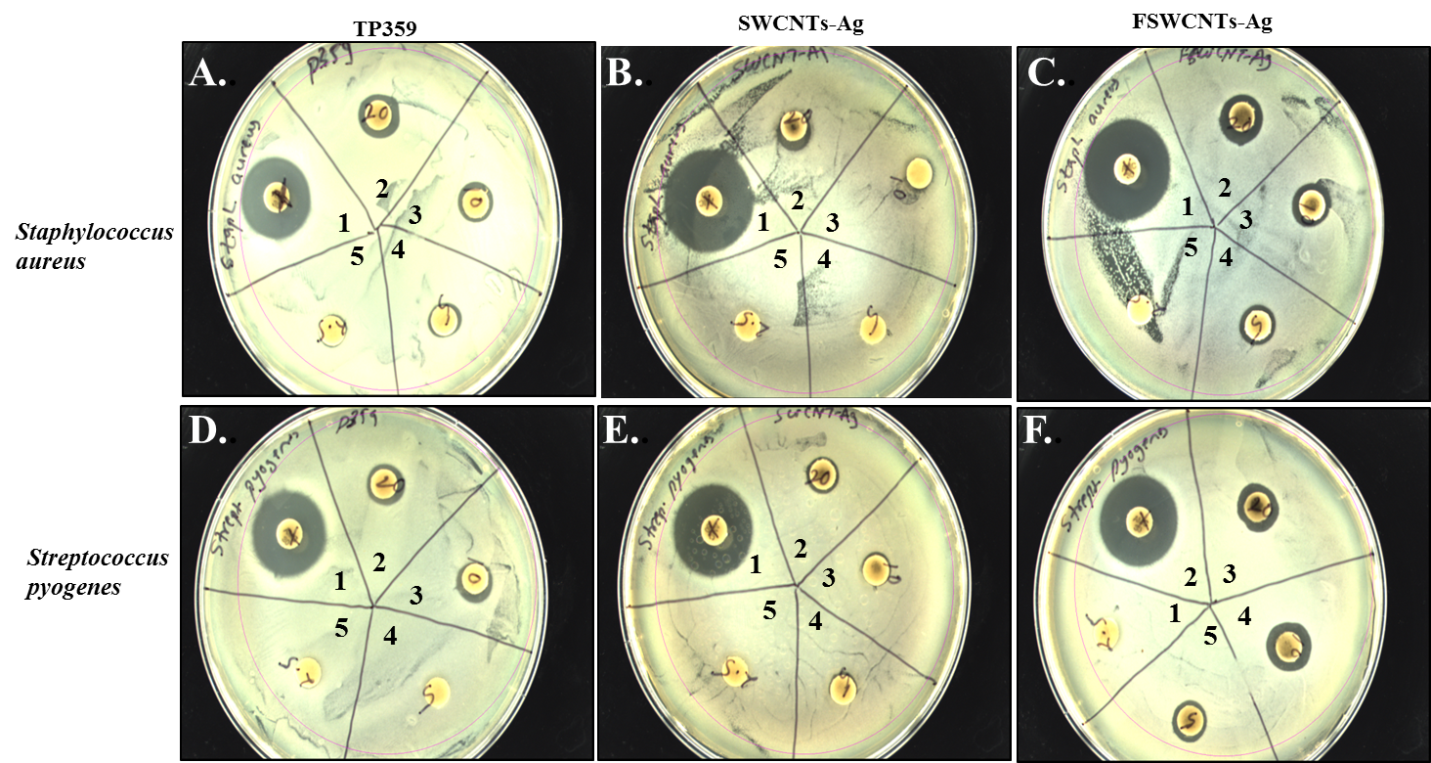
Figure S3.** **Kirby–Bauer disc diffusion assay against gram- positive pathogens.** The MIC concentrations of TP359, SWCNTs-Ag and FSWCNTs-Ag and the broad spectrum antibiotic amoxicillin–clavulanic acid (30 µg) was added to the discs and the can be observed for both the gram- negative pathogens such as (a-c) *Escherichia coli* and (d-f) *Salmonella* Typhimurium. The numbers indicated represents the concentrations of TP359, SWCNTs-Ag and FSWCNTs-Ag; 1: antibiotic control; 2: 20 µg/mL; 3: 10 µg/mL; 4: 5 µg/mL; 5: 2.5 µg/mL.
